# Cellular senescence mediated by p16^INK4A^-coupled miRNA pathways

**DOI:** 10.1093/nar/gkt1096

**Published:** 2013-11-09

**Authors:** Marita G. Overhoff, James C. Garbe, James Koh, Martha R. Stampfer, David H. Beach, Cleo L. Bishop

**Affiliations:** ^1^Centre for Cutaneous Research, Blizard Institute, Barts and the London School of Medicine and Dentistry, Queen Mary University of London, 4 Newark Street, London, E1 2AT, UK, ^2^Life Science Division, Lawrence Berkeley National Laboratory, Berkeley, CA 94720, USA and ^3^Division of Surgical Sciences, Department of Surgery, Duke University Medical School, Durham, NC 27710, USA

## Abstract

p16 is a key regulator of cellular senescence, yet the drivers of this stable state of proliferative arrest are not well understood. Here, we identify 22 senescence-associated microRNAs (SA-miRNAs) in normal human mammary epithelial cells. We show that SA-miRNAs-26b, 181a, 210 and 424 function in concert to directly repress expression of Polycomb group (PcG) proteins CBX7, embryonic ectoderm development (EED), enhancer of zeste homologue 2 (EZH2) and suppressor of zeste 12 homologue (Suz12), thereby activating p16. We demonstrate the existence of a tight positive feedback loop in which SA-miRNAs activate and re-enforce the expression of other SA-miRNA members. In contrast, PcG members restrain senescence by epigenetically repressing the expression of these SA-miRNAs. Importantly, loss of p16 leads to repression of SA-miRNA expression, intimately coupling this effector of senescence to the SA-miRNA/PcG self-regulatory loop. Taken together, our findings illuminate an important regulatory axis that underpins the transition from proliferation to cellular senescence.

## INTRODUCTION

With the exception of pluripotent embryonic stem cells, normal human cells *in vitro* (1) and *in vivo* (2,3) invariably undergo progressive cellular senescence, associated with the eventual cessation of cell division. This state of proliferative arrest is induced by both endogenous and external influences that ultimately converge on either or both of the p16^INK4a^/retinoblastoma (p16/pRb) or p14^ARF^ (p14)/p53 pathways. The binding of p16 to the cyclin-dependent kinase 4–6/cyclin D complex inhibits the phosphorylation of pRb family proteins, causing a G1 cell cycle arrest. In contrast, p14 inactivates MDM2, leading to stabilization of p53 and induction of the p21^Cip1/Waf1^ cyclin inhibitor. The relative involvement of these two tumour suppressor networks is known to be dependent on the cellular context (4). However, both p16 and p14 are encoded by the highly complex INK/alternate reading frame (ARF) locus (9p21.3), which also includes p15^INK4b^ and the long non-coding RNA, ANRIL. This locus is associated with many ageing-associated diseases, such as cancer, type 2 diabetes (5–7) and cardiovascular disease (8,9).

There is now growing evidence that senescence is intimately linked to the loss of regenerative potential and diminished function observed in ageing tissues. The gradual accumulation of p16 expression during physiological ageing (3,10) and several ageing-associated diseases directly implicates this well-established effector of senescence in the ageing process. Senescent cells that can display a pro-inflammatory senescence-associated secretory phenotype (11) may play a role in the chronic pro-inflammatory characteristic of age-related pathologies. Accordingly, the mechanisms of p16 activation both *in vitro* and *in vivo* are of great interest. The future challenge lies not only in identifying the regulators that drive cellular senescence but also in unravelling the upstream factors that control these elements.

Epigenetic mechanisms perform an important role in the initiation and maintenance of cellular senescence. Among these, the Polycomb group (PcG) proteins play a role in genomic imprinting, acting as determinants of cell fate and stem-cell renewal. Polycomb Repressive Complex 2 (PRC2), with its core subunits embryonic ectoderm development (EED), enhancer of zeste homologue 2 (EZH2) and suppressor of zeste 12 homologue (SUZ12), mediates histone H3 lysine 27 methylation (H3K27Me3), typically via the histone methyltransferase EZH2. This epigenetic mark is recognized and enforced by PRC1, which contains BMI1 (BMI1 polycomb ring finger oncogene), CBX7 (chromobox homologue 7) and RING1B (ring finger protein 2). The stable ectopic expression of BMI1 (12), CBX7 (13) or CBX8 (chromobox homologue 8) (14) has been shown to delay cellular senescence by direct repression of p16. An important corollary of the progressive activation of p16 expression during cellular senescence is the gradual decline in the levels of BMI1 and CBX7 bound to the INK/ARF locus (12,13,15). PcG dysregulation is associated in tumourigenesis (16). There is now mounting genetic evidence that mutations, deletions and truncations in the Polycomb components, such as EZH2, occur in a broad range of human cancers. In addition, inactivating somatic mutations of the H3K27 demethylase UTX have been discovered in oesophageal and renal cancers and in multiple myeloma (17).

MicroRNAs (miRNAs) are emerging as regulators of a broad range of cellular functions, including stem cell self-renewal and proliferation (18). These endogenous non-coding RNAs negatively regulate gene expression by targeting mRNAs, predominantly via their 3′UTR, for degradation or translational repression (19). Since the discovery of *lin-4* regulation of *C. elegans* lifespan (20), a number of miRNAs have been identified that play a role in ageing, in both *in vivo* and *in vitro* studies. For example, miR-34a accumulates during senescence in a range of cell types, including the endothelial cells thought to contribute to cardiovascular disease (21–23). This mirrors the gradual accumulation of miR-34 during organismal ageing (24–26). Despite these recent advances, it remains unclear how miRNAs are regulated during cellular senescence, and few of their gene targets have been identified.

Here, we sought to systematically identify miRNA regulators of p16-mediated cellular senescence in normal human mammary epithelial cells (HMECs), and to uncover the cellular pathways through which they act. The cell cycle arrest mediated by p16 in these cells occurs after approximately 50 population doublings from passage 2, does not invoke the p14/p53 axis, and is independent of telomere length (11). We report that combined miRNA screening and miRNA profiling revealed a subset of senescence-associated miRNAs (SA-miRNAs) that act in concert to repress multiple members of the PcG complexes. We further demonstrate that PcG members function to enable cell proliferation by epigenetically repressing the expression of SA-miRNAs. The onset of cellular senescence disrupts this equilibrium and SA-miRNA expression is stimulated. The targeted repression of PcG members propagates the senescence-associated signature and, as a direct consequence, PcG-mediated repression of p16 is relieved and the cellular senescence is activated. Finally, we find that p16 unites these layered feedback loops, ensuring that p16-mediated proliferative arrest is imposed through positive self-reinforcement.

## MATERIALS AND METHODS

### Cells and reagents

Normal finite lifespan HMECs were obtained from reduction mammoplasty tissue of a 21-year-old individual, specimen 184, and were cultured as previously described (11). Presenescent cells at Passage 6 (P6) were used for the miRNA reconstruction, screening and follow-up miRNA studies, unless otherwise stated. Normal finite lifespan human mammary fibroblasts were obtained from reduction mammoplasty tissue of a 16-year-old individual, specimen 48, and were cultured as previously described (27).

### High-content miRNA screening

The miRNA screen was performed using the Human miScript miRNA Mimic Set (V11.0, Qiagen), together with control small interfering RNAs (siRNAs) targeting Cyclophilin B (Dharmacon), CBX7 (Ambion), and p16 (QIAGEN). HMECs at P6 were reverse transfected with 60 nM miRNA in 384-well format using HiperFect (QIAGEN), in triplicate. Plates were incubated for 46 h, medium was changed and fixed/stained 72 h later with Moαp16 JC2, GtαMo AlexaFluor488 (Invitrogen), 4′,6-diamidino-2-phenylindole and Cell Mask (Invitrogen), as previously described (28). High-content images were acquired with the IN Cell 1000 automated microscope (GE) at 10× magnification, and analysis was performed using the Developer Analysis software (GE). Following the screen, the hits were retested in triplicate.

### miRNA microarray

miRNAs were isolated from HMECs at Passage 6 (P6) and Passage 10 (P10) using the miRNeasy Kit (Qiagen) according to the manufacturer’s instructions. The quality of the total RNA was verified by an Agilent 2100 Bioanalyzer profile. One microgram of total RNA from biologically independent triplicates (HMEC P6 and P10) and a common reference were labelled with Hy3™ and Hy5™ fluorescent label, respectively, using the miRCURY™ LNA Array power labelling kit (Exiqon, Denmark) according to the manufacturer’s instructions. The Hy3™-labelled samples and an Hy5™-labelled reference RNA sample were mixed pair-wise and hybridized to the miRCURY™ LNA Array version 11.0 (Exiqon, Denmark), which contains capture probes targeting all miRNAs for human, mouse or rat registered in the miRBASE version 13.0. The hybridization was performed according to the miRCURY™ LNA Array manual using a Tecan HS4800 hybridization station (Tecan, Austria). The miRCURY™ LNA Array microarray slides were scanned using the Agilent G2565BA Microarray Scanner System (Agilent Technologies, Inc., USA), and the image analysis was carried out using the ImaGene 8.0 software (BioDiscovery, Inc., USA). The quantified signals were background-corrected (Normexp with offset value 10) (29) and normalized using the global Lowess (LOcally WEighted Scatterplot Smoothing) regression algorithm.

### miRNA and antigomiR transfections

HMECs were transfected with 60 nM miRNA or 90 nM antigomiR (anti-miRNA) in 384-well plates using HiperFect (Qiagen), and the protocol described above for ‘High-content miRNA Screening’ was followed.

### Quantitative reverse transcriptase-polymerase chain reaction

Methodology for quantitative reverse transcriptase-polymerase chain reaction (qRT-PCR) has been described previously (28).

#### Retroviral stable cell lines

HMEC P5 was transduced with the RasER construct and Ras expression induced as described previously (30). Cells were harvested 48 h post Ras induction for qRT-PCR analysis.

#### Luciferase assays

A list of the primers used to generate the 3′UTR firefly luciferase constructs for CBX7, EED, EZH2 and Suz12, together with the mutagenesis primers, can be found in Supplementary Table S1. HEK293T and HeLa cells were reverse transfected with individual SA-miRNAs, 3′UTR luciferase and Renilla constructs. The firefly and Renilla luciferase activities were measured 48 h post transfection using the Dual-Luciferase Reporter Assay system (Promega). Values are shown relative to a negative control. Luciferase activities of <80% were classified as knockdown.

#### Chromatin immunoprecipitation

Chromatin immunoprecipitation (ChIP) experiments were performed as described previously (28). The primers used for the INK/ARF locus have previously been published (15). The primers used for the SA-miRNA loci can be found in Supplementary Table S4.

## RESULTS

### Identification of SA-miRNAs

**Figure 1.**
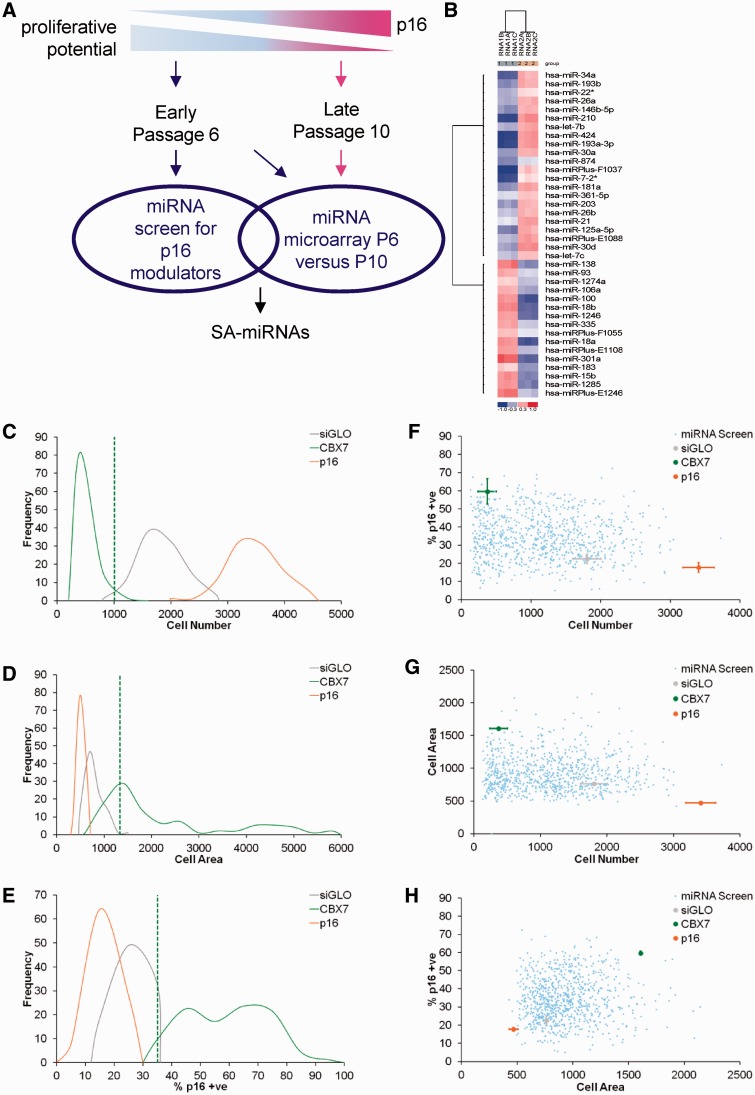
miRNA library screen and miRNA expression profiling identify SA-miRNAs. (**A**) Schematic representation of the strategy to identify SA-miRNAs. (**B**) Heatmap diagram of the results of two-way hierarchical clustering of miRNAs for HMEC P6 and HMEC P10. The clustering was performed on log2 (Hy3/Hy5) rations, which passed the filtering criteria on variation across samples. The relative expression level of each miRNA across all samples is shown: red represents an expression level above the mean and blue expression lower than the mean. (**C–E**) Frequency distributions of (C) cell number, (D) cell area and (E) percentage of p16 positive cells (% p16 +ve) following transfection with siGLO (grey), CBX7 (green) or p16 siRNA (orange) obtained at the time of the miRNA screen for modulators of p16-mediated cellular senescence. Cut-off values for each measure that were used to classify SA-miRNAs (green dotted lines). (**F–H**) Scatter plots illustrating phenotypic criteria data for each control siRNA as per (C–E) and the miRNA screening data (blue). (F) Cell number versus percentage of p16 positive cells (% p16 +ve), (G) cell number versus cell area and (H) cell area versus percentage p16 positive cells.

To identify miRNAs that participate in cellular senescence (SA-miRNAs), we performed an unbiased functional screen for miRNAs that modulate p16 in normal HMECs and cross-referenced these data with microarray expression profiling of endogenous miRNAs that alter during spontaneous cellular senescence (Figure 1A). Using an approach similar to a previously described genome-wide siRNA screen for modulators of p16 (28), we transfected actively proliferating HMECs (Passage 6, P6) in 384-well plates with 837 miRNAs (Sanger version 11.0) at 60 nM. siRNA targeting *siGLO* (‘cyclophilin B’; *PPIB*) served as a negative control, whereas siRNAs against *CBX7* and *p16* acted as positive controls. Using previously defined phenotypic criteria (28) (see ‘Materials and Methods’ section), we assigned cut-off values to define miRNA hits based on reduced cell number, increased cell area and an increase in the percentage of p16 positive cells. The raw screening data and quantitation of each phenotypic criterion are shown (Figure 1C–H). This strategy revealed 62 miRNAs that can activate p16 and induce senescence in normal proliferating HMECs.

To identify miRNA signatures associated with spontaneous cellular senescence, actively proliferating HMECs (HMEC P6) and senescent HMECs (HMEC P10) were analysed by miRNA microarray (Figure 1A). A total of 38 miRNAs were differentially expressed between the two samples (Bonferroni *P* < 0.05): 16 miRNAs were downregulated and 22 miRNAs upregulated during spontaneous cellular senescence (Figure 1B).

By cross-referencing the miRNAs that change in abundance during spontaneous cellular senescence with those that emerged from the functional screen, we discovered that each of the 22 miRNAs that were upregulated during spontaneous cellular senescence scored as positive modulators of p16. These included miR-34 and let-7 family members (see ‘Introduction’ section). The remaining 40 miRNAs that scored positive in the functional screen but which were not classified as SA-miRNAs were not expressed in actively proliferating or senescent HMECs. It is, therefore, possible that this subset miRNAs may play a role in cellular senescence in other cell types or organs. We selected SA-miRNAs-26b, 181a, 210 and 424 for further investigation based on a combined ranking of fold change during senescence and their predicted miRSVR score for PcG members (see later). The expression of each of these SA-miRNAs is induced during p16-mediated cellular senescence, and they are individually potent inducers of the same phenotype.

**Figure 2.**
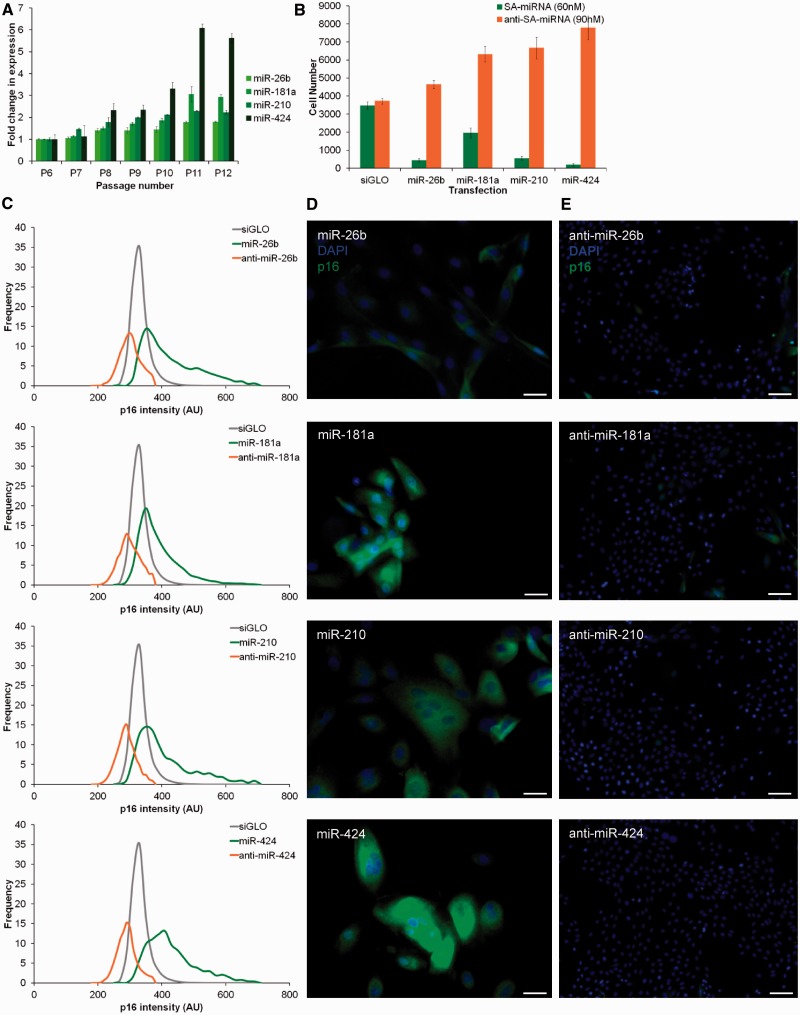
SA-miRNAs function during, and promote, cellular senescence by inducing p16. (**A**) SA-miRNA expression during cellular senescence in normal finite-lifespan HMECs from P6 to P12. (**B**) Cellular proliferation 5 days following transfection of HMEC P6 with the indicated SA-miRNA (60 nM; green) or anti-SA-miRNA (90 nM; orange). (**C**) Frequency distribution of p16 intensity following transfection with siGLO negative control (grey), the corresponding SA-miRNA (green) or anti-SA-miRNA (orange). (**D, E**) HMECs stained with 4′,6-diamidino-2-phenylindole (blue) and αp16 (green) following transfection with (D) SA-miRNA or (E) anti-SA-miRNA. Assays were performed in triplicates, and means ± SD from three independent experiments are shown.

We further validate these four SA-miRNAs. Consistent with the microarray data, RT-PCR analysis showed that the individual SA-miRNAs became progressively elevated as the HMEC culture underwent cellular senescence (Figure 2A). In the case of SA-miRNA-26b and SA-miRNA-181a, the levels of elevation did not exceed a 2-fold increase at P10. However, by P12, SA-miRNA-26b had increased by 1.81-fold and SA-miRNA-181a by 2.93-fold. SA-miRNA-424 exhibited a 5.62-fold increase at P12 relative to P6. The differences in the degree of increase in expression can be explained by the relative levels of expression between the four miRNAs at P6, e.g. SA-miRNA-424 is relatively less abundant than SA-miRNA-26b at this stage. In addition, transient overexpression of each individual SA-miRNAs reduced cell proliferation and elevated p16 levels (Figure 2B–D), validating the results of the miRNA screen. To investigate the potential role of SA-miRNAs in actively proliferating HMECs (HMEC P6), we quenched the low levels of SA-miRNA expression using antogomiRs (antimiRs). The introduction of antimiR-26b, 181a, 210 or 424, each increased cell proliferation (Figure 2B), reduced cell area and reduced p16 levels (Figure 2C and E). Thus, these particular SA-miRNAs apparently play a restraining role at all stages of normal HMEC expansion, under the described conditions of culture.

### SA-miRNAs are elevated during fibroblast senescence

**Figure 3.**
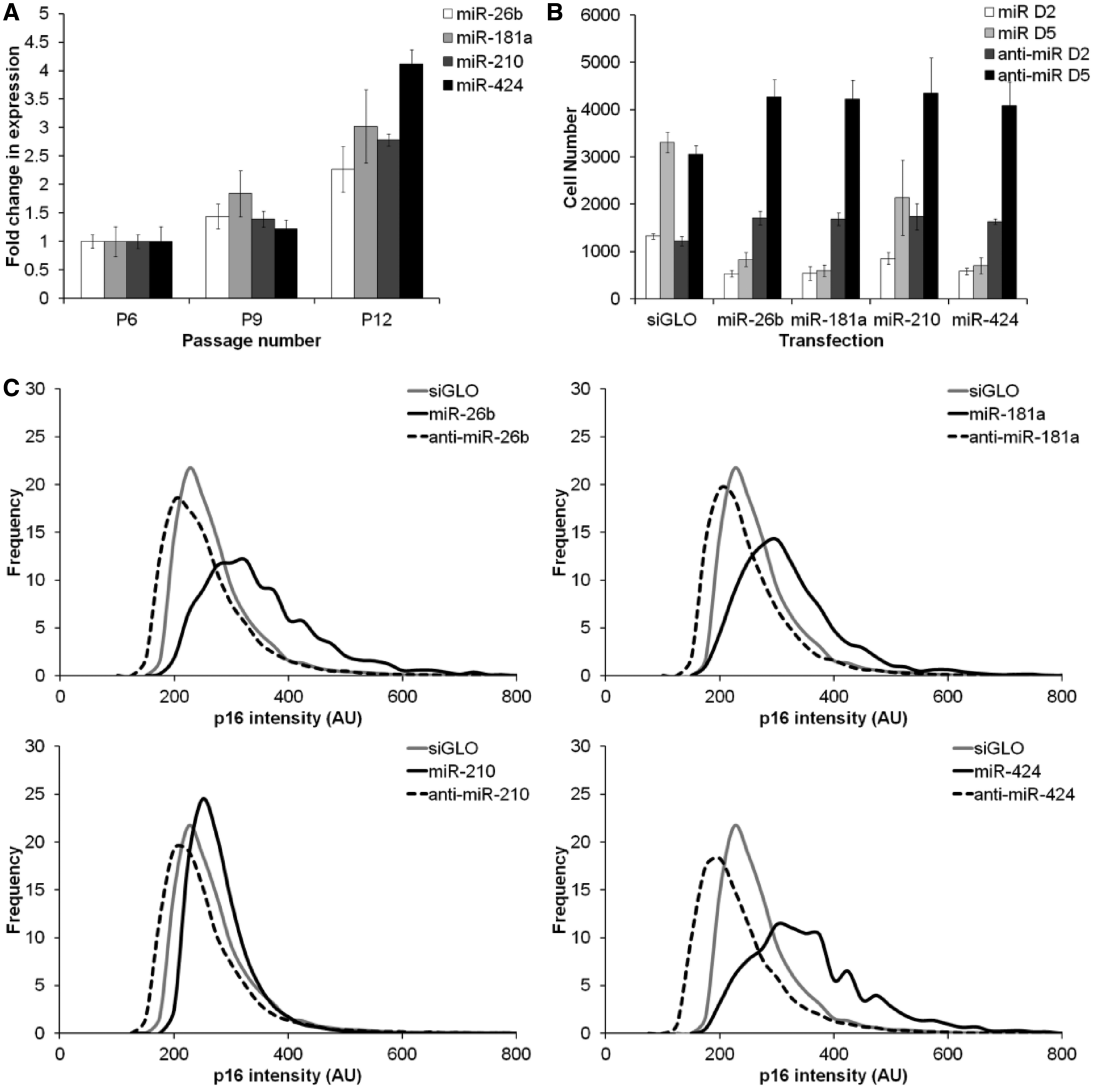
SA-miRNAs function during, and promote, cellular senescence by inducing p16 in fibroblasts. (**A**) SA-miRNA expression during cellular senescence in human fibroblasts from P6 to P12. (**B**) Cellular proliferation following transfection of fibroblasts P6 with the indicated SA-miRNA (60 nM; white, light grey) or anti-SA-miRNA (90 nM; dark grey, black) at day 2 (D2) and day 5 (D5) post transfection. (**C**) Frequency distribution of p16 intensity 5 days following transfection with siGLO negative control (grey), the corresponding SA-miRNA (solid black) or anti-SA-miRNA (dashed black). Assays were performed in triplicates, and means ± SD from three independent experiments are shown.

Next, we sought to determine whether the SA-miRNAs simply played a role in the epithelial senescence programme, or whether they had a broader role in senescence activation in human fibroblasts. Previous work has shown that HMECs at P6 have very few luminal epithelial cells present (31); however, the use of fibroblasts also enabled us to specifically question whether the SA-miRNA changes observed were a result of the loss of this small fraction with serial passage. First, we examined the expression profile of these four SA-miRNAs during serial passage. RT-PCR analysis confirmed a gradual increase in the expression levels of each SA-miRNA with increase in population doublings in fibroblasts (Figure 3A). In addition, transient overexpression of each individual SA-miRNAs in fibroblasts also reduced cell proliferation and increased p16 levels (Figure 3B–C). Transfection with the corresponding antimiRs generated a modest increase in cell proliferation and a decrease in p16 levels. These results confirm that the changes in SA-miRNA expression observed during HMEC senescence are not simply a consequence of a shift in the proportions of luminal and myoepithelial cells between early and late passages. They also verify that a similar relationship between p16 and the SA-miRNAs exists in both HMECs and fibroblasts.

### SA-miRNAs directly and coordinately inhibit expression of PcG genes

To explore the cellular pathways through which the SA-miRNAs might act, we performed target prediction analysis using miRanda and a stringent miRSVR threshold of less than −0.4 (32). This revealed predicted SA-miRNA binding within the 3′UTRs of multiple known transcriptional repressors of the p16 locus. The analysis is presented as a heatmap of the miRSVR score for each of the SA-miRNA targets (Supplementary Figure S1A). A heatmap generated using a control group of miRNAs for these same targets is also shown (Supplementary Figure S1B). These endogenously expressed control miRNAs do not fluctuate during cellular senescence and are not family members of the SA-miRNAs.

**Figure 4.**
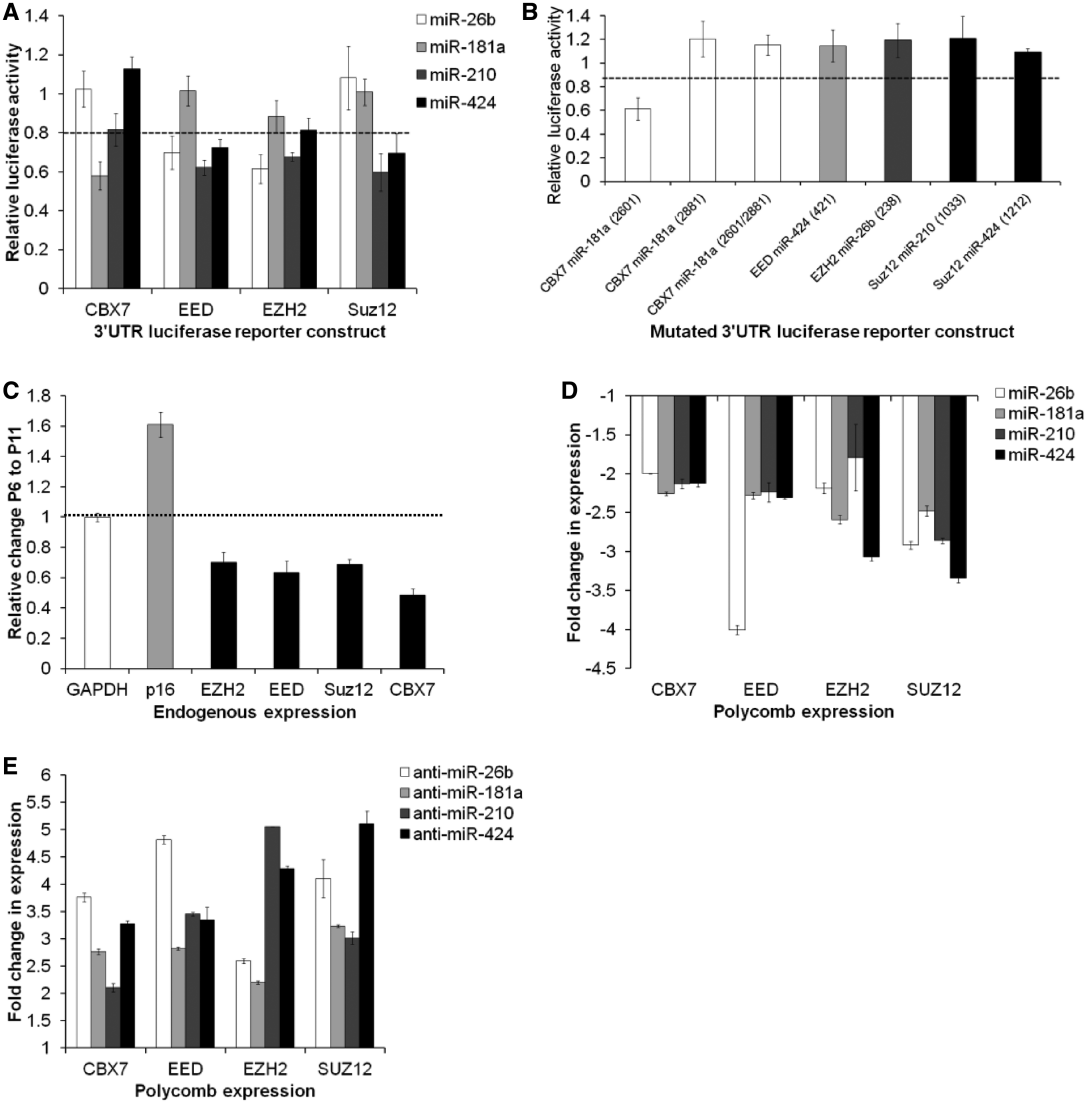
SA-miRNAs directly and coordinately regulate PcG genes. (**A**) miRSVR score analysis predicts potential binding sites for SA-miRNAs in the 3′UTR of multiple PcG mRNAs (see Supplementary Figure S1). Luciferase reporter assays with wild-type 3′ UTR constructs of CBX7, EED, EZH2 or Suz12 demonstrate that SA-miR-26b, 181a, 210 and 424 repress PcG protein activity. (**B**) Luciferase reporter assays with mutated 3′UTR constructs as described in Supplementary Table S1. Values for mock transfections were normalized to 1. (**C**) PcG protein expression declines during cellular senescence. (**D, E**) qRT-PCR quantitation of PcG expression following transfection of HMEC P6 with the indicated (D) SA-miRNA or (E) anti-SA-miRNA. Assays were performed in triplicates, and means ± SD from three independent experiments are shown.

Among the predicted targets of the SA-miRNAs were several PcG genes. To establish whether the SA-miRNAs directly target these INK/ARF transcriptional repressors, we generated luciferase reporter constructs containing the full-length human 3′UTR of CBX7 (PRC1), EED, EZH2 and Suz12 (PRC2). Co-transfection of each of the four 3′UTR constructs with each individual SA-miRNA-26b, 181a, 210 or 424 was performed. These experiments revealed the following: SA-miRNA-26b inhibited luciferase activity from the EED 3′UTR and EZH2 3′UTR constructs; SA-miRNA-181a inhibited CBX7; SA-miRNA-210 inhibited EED, EZH2 and Suz12; and SA-miRNA-424 inhibited EED and Suz12 (Figure 4A; *P* < 0.05). Given that base pairing between the miRNA seed sequence and its target mRNA is required for repression, we introduced selected point mutations to disrupt the predicted complementarity for each SA-miRNA target sites within the CBX7, EED, EZH2 and Suz12 3′UTR constructs (Supplementary Table S1). The target prediction programme miRWalk (33) was also used at this stage and confirmed that at least six different established programmes support each of the miRSVR prediction (Supplementary Table S2). Mutation of the individual SA-miRNA binding sites within the 3′UTR of EED, EZH2 and Suz12 abolished the repression by each of the SA-miRNA mimics (Figure 4B). Of the two predicted SA-miRNA-181a sites within the 3′UTR of CBX7, only the site at position 2881 is necessary for the downregulation of CBX7 expression. A diagrammatic summary of the findings is presented in Supplementary Figure S3. Interestingly, a downregulation of CBX7, EED, EZH2 and Suz12 mRNA levels is also observed during cellular senescence (Figure 4C). Taken together, these observations suggest that the four SA-miRNAs identified in this study act coordinately to suppress expression of multiple PcG members.

Next, we tested whether the SA-miRNAs repress endogenous PcG expression in HMECs. Transient overexpression of SA-miRNA-26b, 181a, 210 or 424 in presenescent HMECs universally inhibited CBX7, EED, EZH2 and Suz12 gene expressions (Figure 4D). Conversely, introduction of individual antimiR relieved SA-miRNA-mediated repression of each PcG members, elevating their expression (Figure 4E). These results suggest that the SA-miRNA drives cellular senescence, in part, by coordinately repressing these known INK/ARF regulators.

### SA-miRNAs, PcG and p16 are interconnected in coupled feedback loops

**Figure 5.**
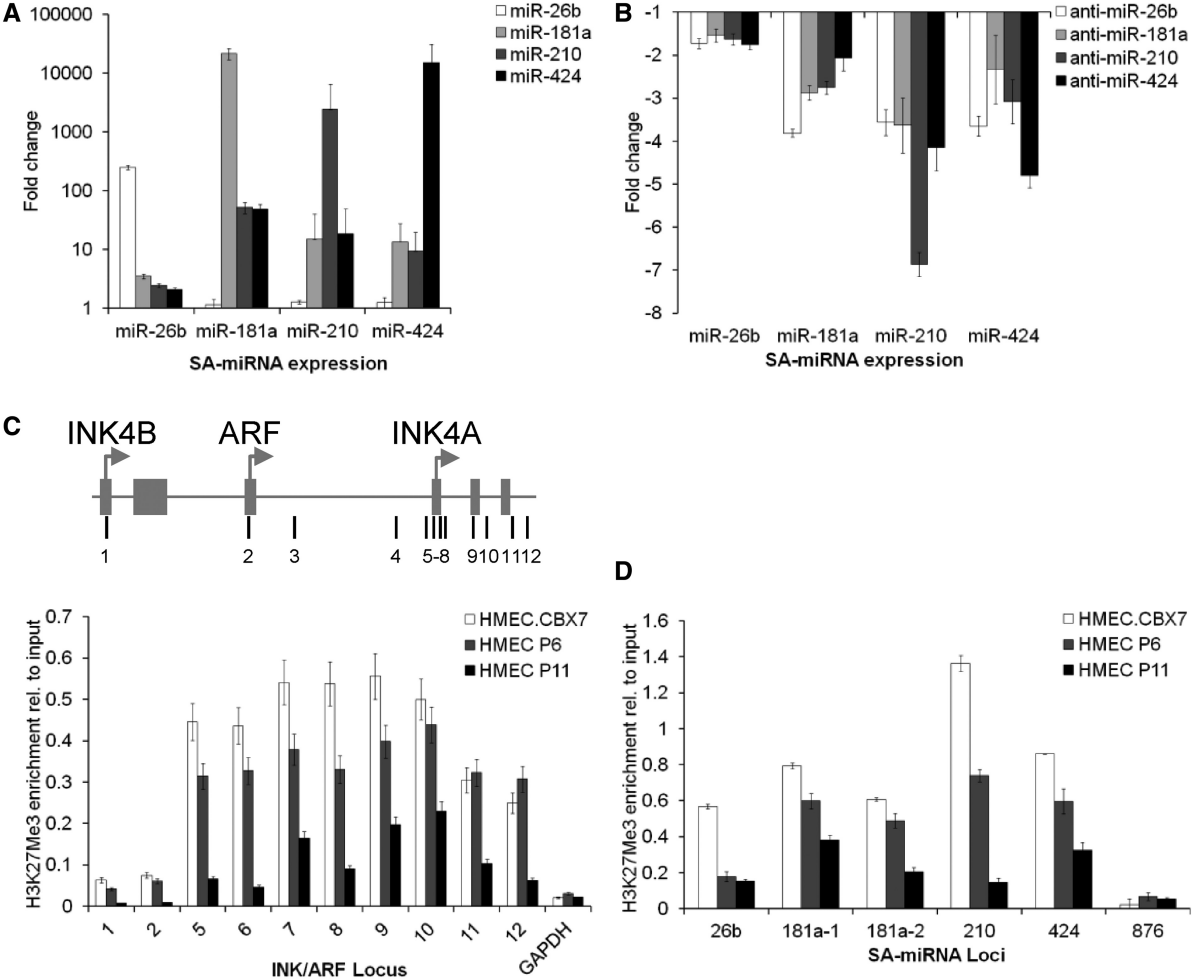
p16, PcGs and the SA-miRNAs are interconnected in coupled feed-back loops. (**A** and **B**) Fold change in SA-miRNA expression following the transfection of HMEC P6 with the indicated (A) SA-miRNA or (B) anti-SA-miRNA. (**C** and **D**) ChIP analysis of H3K27Me3 at (C) the indicated regions of the INK/ARF locus, and (D) the genomic loci of SA-miRNAs following stable overexpression of CBX7 (HMEC.CBX7) in HMEC P6 and HMEC P11 cells. ChIP analysis of GAPDH and miR-876 serves as negative controls. A map to show where the primers align is given for INK/ARF locus in the top (C) and for the SA-miRNAs in Supplementary Table S4. Assays were performed in triplicates, and means ± SD from three independent experiments are shown.

To further elaborate the relationship between each SA-miRNA, we examined the impact of modulating the levels of individual SA-miRNAs on other SA-miRNA members. Introduction of SA-miRNA-181a prompted an elevation of SA-miRNA-26b, 210 and 424 levels. Similar crosstalk was observed following the introduction of SA-miRNA-210 or 424 (Figure 5A). In contrast, overexpression of SA-miRNA-26b failed to generate a similar increase in SA-miRNA-181a, 210 or 424. However, antimiR exposure revealed that loss of SA-miRNA-26b generated downregulation of SA-miRNA-181a, 210 and 424. Comparable results were observed for antimiR-181a, 210 and 424 (Figure 5B). Given that each of these SA-miRNAs is encoded on a different chromosome (Supplementary Table S3), these findings suggest the existence of positive feedback loops in which individual SA-miRNAs transactivate the expression of other SA-miRNA member (Supplementary Figure S2). We propose that this self-propagating loop acts to ensure the coordinated repression of multiple PcG members by the SA-miRNAs. As a direct consequence, the epigenetic repression of p16 is relieved and the senescence phenotype imposed.

### SA-miRNAs are silenced by PcG members

Given that SA-miRNAs are able to transactivate one another, and their co-ordinated activation is required for the senescent phenotype, we hypothesized that SA-miRNA expression is regulated by a common epigenetic mechanism. Previous work has shown that the genetic locus encoding miR-181a is epigenetically repressed by PcG members in the cancer cell line DU145 (34), and previously published ChIP-seq studies for H3K27Me3 in HMECs show enrichment at the hsa-miR-424 locus (ENCODE). The inverse relationship between PcG gene expression and SA-miRNA levels during senescence led us to ask whether PcG members conversely act to transcriptionally silence SA-miRNAs. To test this, we performed ChIP assays with anti-H3K27Me3 in presenescent and senescent HMECs (HMEC P6 versus HMEC P11). We used the INK/ARF locus as a positive control for these assays, as the PcG-mediated repression of p16 is very well established (15). As expected, we observed decreased H3K27Me3 occupancy within the INK/ARF locus during cellular senescence (Figure 5C). Likewise, a marked decrease in H3K27Me3 occupancy at loci for SA-181a-1, 181a-2, 210 and 424 was also observed during cellular senescence (Figure 5D). The locus for miRNA-876 served as a negative control and showed no change in H3K27Me3 occupancy. Furthermore, stable overexpression of CBX7 generated a marked increase in H3K27Me3 occupancy at both the INK/ARF locus and at each SA-miRNA genomic locus. These data point to the existence of a negative feedback loop through which PcG members act to repress SA-miRNA expression in presenescent cells.

### p16 coupled to SA-miRNA and PcG regulatory mechanism

**Figure 6.**
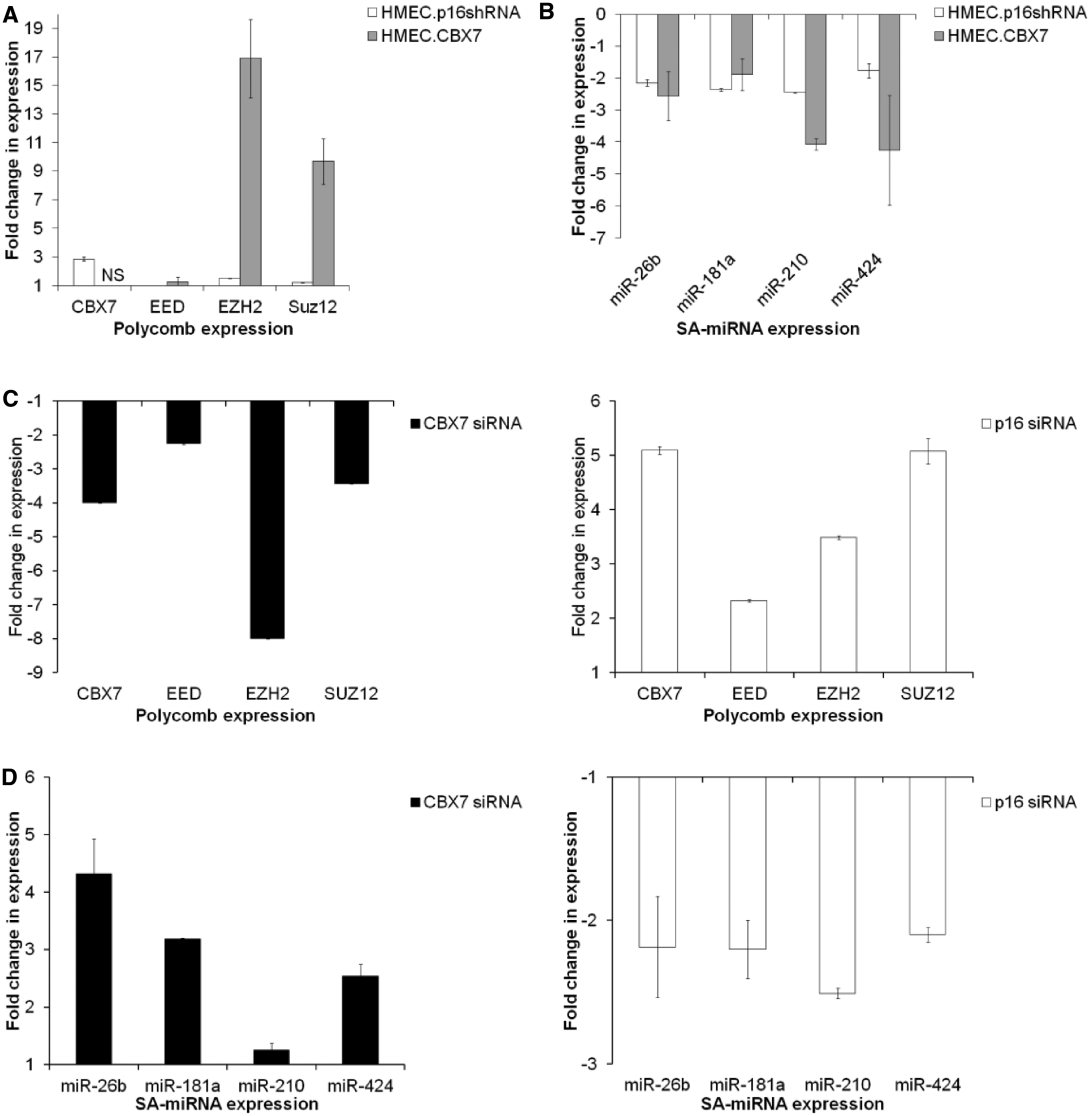
p16 coupled to SA-miRNA and PcG regulatory mechanism. (**A** and **B**) Fold change in (A) PcG and (B) SA-miRNA expression following stable knockdown of p16 expression (HMEC.p16shRNA) or stable CBX7 overexpression (HMEC.CBX7). Data are normalized to HMEC.Vector control. NS = not shown. The fold change in CBX7 expression in the HMEC.CBX7 relative to HMEC.Vector was +1294 ± 58.96. (**C** and **D**) Fold change in (C) Polycomb members and (D) SA-miRNA expression following transient knockdown of CBX7 (green, left) or p16 expression (orange, right). Data expressed relative to the siGLO negative control. Assays were performed in triplicates, and means ± SD from three independent experiments are shown.

SA-miRNAs act to drive the activation of p16 expression and promote senescence. We hypothesized that, in turn, p16 might act positively to fortify its own expression. Consistent with this notion, stable knockdown of p16 (HMEC.p16shRNA) generated a relative increase in PcG member expression, particularly in the case of CBX7 (Figure 6A). Concordantly, SA-miRNA levels were reduced (Figure 6B). One interpretation of these data is that loss of p16 acts to extend proliferative lifespan by delaying the transition to SA-miRNA activation and PcG decay. Interestingly, overexpression of CBX7 led to similar findings. As expected, CBX7 overexpression generated a dramatic increase in CBX7 levels (1294 ± 58.96, data not shown). Although EED levels remained virtually unchanged, the relative levels of EZH2 and Suz12 expression increased (Figure 6A), suggesting further positive cross-talk between PcG members. Similar to p16 knockdown, overexpression of CBX7 resulted in the reduction of SA-miRNA levels (Figure 6B). These data would further support the hypothesis that maintenance of PcG member expression is crucial for suppression of SA-miRNA expression and restraining senescence. Next, we examined the impact of the transient loss of p16 on PcG and SA-miRNA expression. Transient transfection of presenescent HMECs with a potent siRNA targeting p16 increased the expression of each PcG member (Figure 6C) and repressed SA-miRNA levels (Figure 6D) to a great degree than the changes observed in HMEC.p16shRNA cells. The siRNA knockdown of CBX7 in this setting reduced the levels of not only CBX7 but also of EED, EZH2 and Suz12, accompanied by an anticipated increase in SA-miRNA levels. These findings suggest that p16 itself can modulate the delicate equilibrium between its own regulators, PcG members and SA-miRNAs. This integrated role of p16 self-reinforcement within the feedback loops uncovered here suggests an additional layer of intricacy in the establishment and maintenance of this complex phenotype.

## DISCUSSION

**Figure 7.**
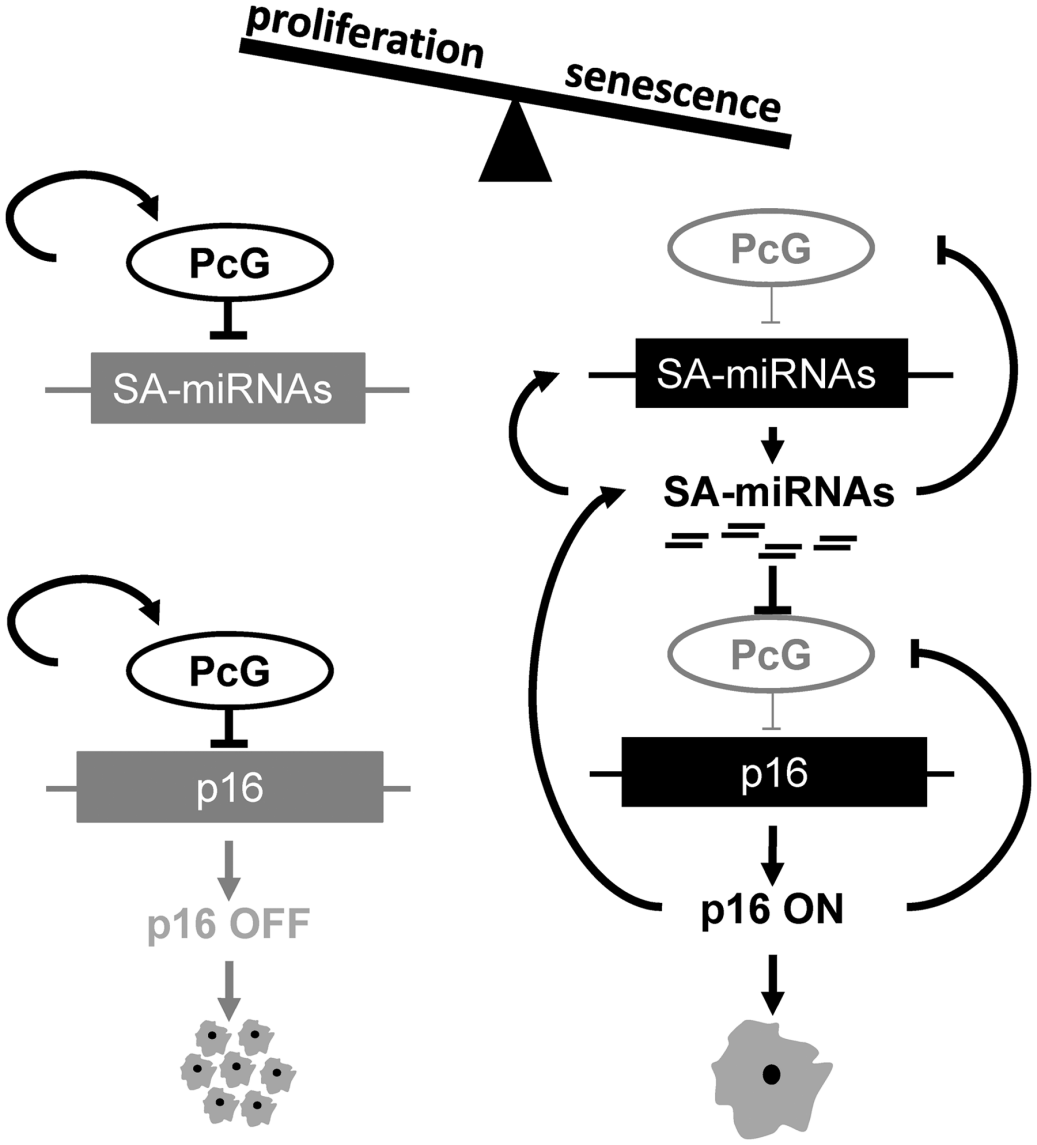
p16-coupled miRNA pathways mediate cellular senescence. PcG members CBX7, EED, EZH2 and Suz12 function to restrain cellular senescence by epigenetically repressing the expression of SA-miRNAs, SA-miRNA-26b, 181a, 210 and 424, as well as p16. CBX7 functions to positively promote the expression of other PcG members. The onset of cellular senescence disrupts this equilibrium, and SA-miRNA expression is stimulated. SA-miRNAs directly target PcG mRNAs for degradation. This, together with SA-miRNA cross-talk, ensures continued expression of the SA-miRNA signature, and as a direct consequence, PcG-mediated repression of p16 is relieved and the senescence programme enforced. p16, in turn, ensures self-reinforcement through a positive feedback loop with SA-miRNAs and a negative feedback loop with PcG members.

Here, we propose a model of coordinated and interconnected regulatory feedback loops that tie SA-miRNA to p16, indirectly through expression of PcG family members (Figure 7). Initially, we identified 22 SA-miRNAs by cross-referencing the outcome of a functional miRNA screen for modulators of p16-mediated senescence with miRNA expression profiling during cellular senescence. We demonstrated that SA-miRNAs-26b, 181a, 210 and 424 drive cellular senescence by directly repressing a panel of PcG members. Each of these four SA-miRNAs repressed endogenous CBX7, EED, EZH2 and Suz12 mRNA levels. This is achieved through a tight positive feedback loop in which individual SA-miRNAs activate and reinforce the expression of other SA-miRNA members. As a consequence, the ectopic expression of any single SA-miRNA activates expression of other SA-miRNA members. This, in turn, leads to the co-ordinated repression of PcG members and the induction of cellular senescence. We also show that PcG members function to restrain cellular senescence by epigenetically repressing the expression of SA-miRNA-181a, 210 and 424. Finally, we propose an additional layer of regulation in which p16 reinforces cellular senescence through a feedback mechanism that promotes SA-miRNA expression, thereby suppressing the PcG-mediated epigenetic repression of both SA-miRNAs and p16.

The original 22 SA-miRNAs identified here included members of the highly conserved miR-34 and let-7 families. The miR-34 family (miR-34a, b and c) increases both during cellular senescence (21,35) and with organismal age in a range of tissues (22) and species (25,36), and acts to inhibit cell proliferation by upregulating the p53/p21 axis (37). Our results suggest that miR-34a may additionally function to regulate cell proliferation through activation of p16. Our work also builds on earlier mouse studies, which observed increased let-7b in mouse embryo firbroblast (MEFs) (38), and confirms that let-7b and let-7c elevation during senescence extends to human epithelial senescence. The decline in neural stem cell function has previously been shown to be driven by let-7b-mediated downregulation of HMGA2, a negative regulator of p16 (39). Together, this suggests a varied range of activities for the SA-miRNAs in cellular senescence, and more broadly in ageing phenotypes.

In this work, we show that overexpression of SA-miRNA-26b, 181a, 210 or 424 induced cellular senescence in both human epithelial cells and fibroblasts, while their ablation represses p16 levels. These similarities between miRNA expression contrast with the general lack of concordance seen in gene transcript analysis between these mammary epithelial cells and fibroblasts (11), and suggests that the SA-miRNAs may be more central to the process of p16-mediated senescence. In support of this, miRNA-26 has previously been shown to be upregulated in ageing MEFs (38) and downregulated in long-lived humans (40). Similarly, miR-181a is elevated during cellular senescence in keratinocytes (41), inducing senescence in part by direct inhibition of p63 and Sirt1 (42). Further, both miRNA-181a and miRNA-34a are downregulated in long-lived calorie-restricted mice (43). Similarly, miRNA-210 has also been shown to increase during cellular senescence in IMR90 fibroblasts, and its overexpression induces the accumulation of reactive oxygen species and DNA damage (44). Finally, the miRNA-424 polycistron is activated in senescent BJ fibroblasts (45) and has been shown to induce a G1 cell cycle arrest in human THP-1 cells (46). Our work unites these studies, and suggests that the four SA-miRNAs studied here may play a similar role in cellular senescence in a broad range of cell types.

We demonstrate that PcG members CBX7, EED, EZH2 and Suz12 are coordinately and directly repressed by SA-miRNA-26b, 181a, 210 and 424. Consistent with this result, a role for miRNA-26a repression of the PRC2 component EZH2 has previously been demonstrated in the context of tumourigenesis and myogenesis (47,48). More recently, miRNA-181a was established as a direct regulator of RING2 (PRC2) (34) and CBX7 (PRC1) in cancer (49). Our results are in agreement with the notion that this fundamental regulatory axis may become hijacked and dysregulated in cancer. Given the coordinated repression of CBX7, EED, EZH2 and Suz12 by SA-miRNAs, our findings indicate that these miRNAs function to target both the initial trimethylation of H3K27 mediated by PRC2, and also to inhibit the chromatin condensation and epigenetic silencing achieved following PRC1 recruitment. Although the mechanism by which PRC2 and PRC1 synchronize their activities is not yet clear, our data support the notion that SA-miRNA-mediated regulation coordinates this process.

We also established a role for PcG members in the epigenetic repression of SA-miRNA transcription, via a direct binding to multiple SA-miRNA loci. In line with this, the miRNA-181a genetic loci were previously shown to be a direct target epigenetic silencing via EZH2-mediated recruitment of H3K27Me3 (34), and our findings further extend this list. Given that the ectopic expression of BMI1 (12), CBX7 (13) or CBX8 (14) has been shown to delay cellular senescence by downregulating p16, we propose that PcG members can lengthen proliferative lifespan by direct epigenetic repression of multiple SA-miRNAs.

Advancing age is a risk factor for most chronic human diseases, including cancer, cardiovascular disease and type 2 diabetes. There are overwhelming data implicating p16 for each of these conditions. More recently, clearance of p16-positive cells was shown to prevent or delay a broad range of age-related phenotypes in a particular progeric mouse model (50), suggesting a direct mechanistic link between cellular senescence, p16 and ageing. It is therefore tempting to speculate that the SA-miRNA/PcG/p16 regulatory feedback loops identified here may also function during chronological ageing.

## SUPPLEMENTARY DATA

Supplementary Data are available at NAR Online.

## FUNDING

The Director, Office of Science, Office of Biological and Environmental Research of the U.S. Department of Energy under Contract No. [DE-AC02-05CH11231 to J.C.G. and M.R.S.]. Funding for open access charge: Medical Research Council, UK

*Conflict of interest statement*. None declared.
